# Dynamic focal retinal arteriolar vasospasm in migraine

**DOI:** 10.4103/0301-4738.73717

**Published:** 2011

**Authors:** Anmar M Abdul-Rahman, Jagjit S Gilhotra, Dinesh Selva

**Affiliations:** Department of Ophthalmology, Manukau SuperClinic, Private Bag 98743, South Auckland Mail Centre, Auckland 6, New Zealand

**Keywords:** Branch retinal artery occlusion, migraine, vasospasm

## Abstract

A 48-year-old man presented following an episode of sudden onset simultaneous inferior altitudinal visual loss in his left eye and visual obscuration with shimmering in the inferonasal quadrant of the right eye. Clinical examination demonstrated left superior hemiretinal artery occlusion and an area of focal dynamic spasm along the right superior temporal branch retinal artery, the arteriolar spastic cycle was about 2 sec in duration. Hematological (including complete blood count, thrombophilia screen, vasculitic screen and serum magnesium), carotid, and cardiac investigations were normal. He was given acetazolamide 500 mg orally, timolol maleate 0.5% eye drops once daily and sublingual amyl-nitrate 0.8 mg, and maintained on felodipine 10 mg/day and aspirin 100 mg/day. The area of focal arteriolar spasm in the right eye resolved over two months. To our knowledge there are no prior reports of photographically documented dynamic focal retinal vascular spasm on a MEDLINE and PUBMED search.

The ocular circulation may be involved in vasospastic syndrome; ocular manifestations of vasospasm include conjunctival vasoconstriction, corneal edema, retinal arterial and venous occlusion, ischemia, amaurosis fugax, anterior ischemic optic neuropathy, and glaucoma.[1] Retinal arteriolar vasospasm is rare, occurring in approximately one in 200 migraine sufferers.[2]

We describe a case of simultaneous bilateral retinal arteriolar spasm in a patient with a long history of migraine. One eye presented with hemiretinal arterial occlusion and the other eye with a dynamic focal retinal arteriolar vasospasm.

To our knowledge there are no prior reports of dynamic focal retinal vascular spasm on a MEDLINE and PUBMED search.

## Case Report

A 48-year-old man presented following an episode of sudden onset simultaneous inferior altitudinal visual loss in his left eye and visual obscuration with shimmering in the inferonasal quadrant of the right eye. There was no headache at the time of presentation. He neither had a preceding aura nor any identified trigger factor.

His past medical history included classic migraine with aura diagnosed at the age of six years. However, he ceased to have headaches at the age of 20 and described subsequent episodes occurring every three to four months for the last year of unilateral visual disturbance occurring in either eye consisting of marching positive scotomata or a shimmering effect lasting about 45-60 min. These episodes had no associated headache and usually involved a different part of the visual field.

**Figure 1:**
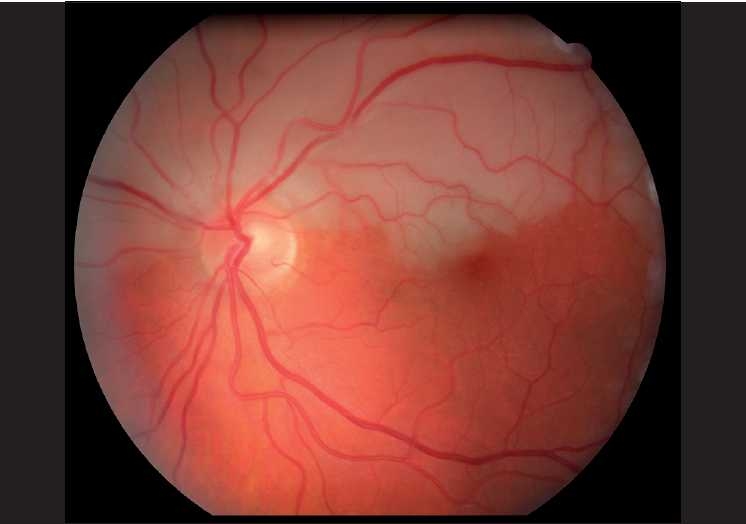
Color fundus photo of the left eye demonstrating left superior hemiretinal artery occlusion

**Figure 2(a-d):**
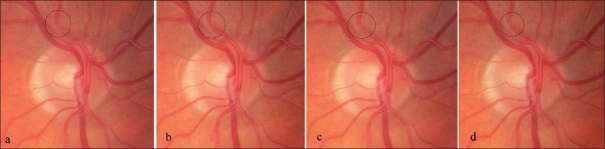
Area of focal dynamic spasm along the right superior temporal branch retinal artery, the arteriolar spastic cycle was about 2 sec in duration. Timing of the vasospastic cycle was performed at a slit-lamp and photographs (a-d) were subsequently taken to represent different phases of the cycle

He was not on any prophylactic treatment for migraine and the last episode was one month prior to presentation. On examination visual acuity was 20/20 bilaterally; he had a left relative afferent pupillary defect and a left inferior altitudinal visual field defect. The right visual field was normal. Left eye fundus examination revealed superior hemiretinal artery occlusion [Fig. 1]. Right eye fundus showed area of dynamic spasm along the superior temporal branch retinal artery, the arteriolar spastic cycle was about 2 sec in duration [Fig. 2a–c]. Timing of the vasospastic cycle was performed at a slit-lamp and photographs were subsequently taken to represent different phases of the cycle.

He was given acetazolamide 500 mg orally, timolol maleate 0.5% eye drops once daily and sublingual amyl-nitrate 0.8 mg, and maintained on felodipine 10 mg/day and aspirin 100 mg/day.

Hematological (including complete blood count, thrombophilia screen, vasculitic screen and serum magnesium), carotid, and cardiac investigations were normal.

The area of focal arteriolar spasm in the right eye ceased dynamic constriction at Day 2 after presentation; resolution of the vasospastic segment was noted at two months follow-up and symptoms correspondingly subsided, there were no changes in the symptoms in the left eye.

## Discussion

Choroidal and optic nerve head circulation involvement in primary vasospastic syndrome has been described by Gasser *et al*..[3] and Flammer *et al*.[1] This is a rare condition and difficult to capture in progress. Our case provides photographic evidence of a dynamic unilateral focal retinal arteriolar stenosis occurring synchronously with a contralateral retinal hemiarterial occlusion.

Vasospastic syndrome is characterized by a hyper-responsiveness of patients with spasm to stimuli like cold or emotional stress. Primary vasospasm occurs in those without an underlying disorder while secondary vasospasm can occur in a variety of autoimmune diseases such as multiple sclerosis, lupus erythematosus, antiphospholipid syndrome, infectious diseases such as AIDS, tumors, drugs, as well as after head injury.[1] Primary vasospasm can affect several organs concurrently or sequentially.

Patients with migraine are more likely to have a vasospastic syndrome.[4] However, the relationship between migraine and vasospastic syndrome has not been fully elucidated in that not all vasospastic patients have migraine, and not all migraine patients have a vasospastic syndrome.[1] In our case also the patient had a history of migraines. Patients with primary vasospastic syndrome are more likely to suffer from cold hands and feet, and low blood pressure, notably at night. Nail fold capillary microscopy and angiography after a cold challenge, and an increased plasma level of endothelin-1 are useful for diagnosis.[1]

Gasser *et al*., reported that patients with vasospastic disorders often had visual field defects.[3] While Guthauser *et al*., observed that such visual field defects could be provoked by cold and improved by calcium channel blockers in patients with vasospastic diathesis but not in normals.[5]

Retinal arterial occlusions are more likely in older patients with arteriosclerosis, however, they have been observed in young patients who have neither risk factors nor signs of arteriosclerosis.[6] These patients are likely to have a vasospastic syndrome.[1] This was also the case in our patient who was middle-aged and had a left retinal artery occlusion and vasospasm. Vasospastic amaurosis fugax with reduced retina perfusion has also been reported.[7]

The exact pathophysiology of focal retinal vasospasm remains unknown, however, several mechanisms have been postulated. Retinal circulation lacks autonomic supply and is controlled by autoregulation determined by the balance of Endothelin-1 (ET-1) and endothelium-derived nitric oxide (NO). ET-1, which increases in all diseases related to vasospasm, contributes to retinal vasoconstriction by inducing vascular hyper-responsiveness to various stimuli rather than being the direct cause of vasospasm.[1,8,9] NO participates in the regulation of ET-1 via a cGMP-related mechanism; a reduced local function of NO in the diseased vascular segment could contribute to enhanced ET-1 production.[1] Prostaglandins also have a complex effect on retinal vasoregulation.[10] Physical (perfusion pressure) and metabolic factors (e.g. PO_2_, PCO_2_, pH and chemical mediators) influence this process.[10]

Our case shows that the retinal vasculature provides a unique window to visualize the dynamic vasospastic process. In addition it demonstrates that primary vasospastic syndrome involving the ocular circulation can follow an aggressive course, and it should be considered as a part of the differential diagnosis in younger patients as should prophylactic treatment with calcium channel blockers in patients with evidence of vasospasm prior to the onset of visual loss or involvement of other organ systems.
